# Cutaneous Squamous Cell Carcinoma of the Head and Neck: Pathological Features and What They Mean for Prognosis and Treatment

**DOI:** 10.3390/cancers16162866

**Published:** 2024-08-17

**Authors:** Uma Ramesh, Elizabeth Chiang, Haleigh Stafford, Jane Buell, Frank Materia, Moran Amit, Dan Yaniv

**Affiliations:** 1 School of Medicine, Baylor College of Medicine, Houston, TX 77030, USA; uma.ramesh@bcm.edu (U.R.); elizabeth.chiang@bcm.edu (E.C.); haleigh.stafford@bcm.edu (H.S.); jane.buell@bcm.edu (J.B.); 2 Department of Otolaryngology—Head and Neck Surgery, University of Kansas Medical Center, Kansas City, KS 66160, USA; fmateria@kumc.edu; 3 Department of Head and Neck Surgery, The University of Texas MD Anderson Cancer Center, Houston, TX 77030, USA

**Keywords:** cutaneous squamous cell carcinoma, prognosis, adjuvant treatment, review

## Abstract

**Simple Summary:**

Cutaneous squamous cell carcinoma (cSCC) of the head and neck, although treatable, can be highly aggressive. Certain pathological features of cSCC can drastically impact prognosis. Therefore, the presence of a few such features is considered in both staging and treating cSCC. In this review, we summarize the current literature regarding pathological prognostic indicators of cSCC, including depth of invasion, surgical margins, perineural invasion, lymphovascular invasion, extranodal extension, tumor grade, tumor subtype, premalignant lesions, and molecular markers. Furthermore, we characterize the impact of each indicator on clinical outcomes of head and neck cSCC, their role in dictating adjuvant therapy for this tumor, and their incorporation or lack thereof into current staging and treatment guidelines.

**Abstract:**

Cutaneous squamous cell carcinoma (cSCC) is one of the most common cancers worldwide, with an incidence that has increased over the past 30 years. Although usually curable with excision, cSCC can become widely metastatic and aggressive with poor outcomes. Whereas the clinical and radiographic extent of any cancer will always guide selection of treatment modality, pathological features of cSCC also play an important role in determining prognosis and, subsequently, the need for further therapy. Therefore, reviewing and summarizing the current literature regarding pathological prognostic indicators of cSCC is essential to improving clinical outcomes. The present literature review yielded depth of invasion, surgical margins, perineural invasion, extranodal extension, lymphovascular invasion, tumor grade, tumor subtype, premalignant lesions, and molecular markers as key prognostic indicators, all with varying recommendations for adjuvant therapy. Notably, some of these factors have not been incorporated into either the American Joint Committee on Cancer staging system (8th edition) or National Comprehensive Cancer Network Clinical Practice Guidelines in Oncology for cSCC. This review highlights a need for further research into these prognostic indicators and their role in determining the need for adjuvant treatment in head and neck cSCC.

## 1. Introduction

Cutaneous squamous cell carcinoma (cSCC) is among the most common cancers in the United States, and its incidence is increasing annually worldwide [1]. cSCC is the most common metastatic skin cancer and is most often located in the head and neck region [2]. Although the vast majority of cSCCs are successfully treated in early stages, cases of progressive disease are often extremely aggressive [2]. Treatment can range from electrodessication and curettage in an outpatient setting to extensive surgery requiring reconstruction, radiotherapy, and systemic agents depending on the extent and location of disease. Certain tumor characteristics portend worse prognoses than others. Some characteristics may be clinically apparent (i.e., large tumor, gross necrosis), whereas others are only evident upon histological examination (i.e., high tumor grade). In some cases, the presence of certain pathological tumor characteristics can have such a profound impact on prognosis that the planned treatment course must be modified [3].

The American Joint Committee on Cancer (AJCC) staging system (8th edition) and National Comprehensive Cancer Network (NCCN) Clinical Practice Guidelines in Oncology for cSCC incorporated some of these pathological factors, such as perineural invasion (PNI), in staging and in selecting treatment modalities, respectively. However, it is necessary to iteratively update NCCN guidelines as newer literature regarding pathological features of cSCC emerges. Therefore, we deemed a literature review appropriate to summarize current findings regarding prognostic indicators for cSCC and their role in determining the need for adjuvant treatment. The aim of this review was to compare recommendations for treatment in the existing literature with those in the 8th edition of the AJCC staging system and current NCCN guidelines for head and neck cSCC.

## 2. Depth of Invasion

**Figure 1:**
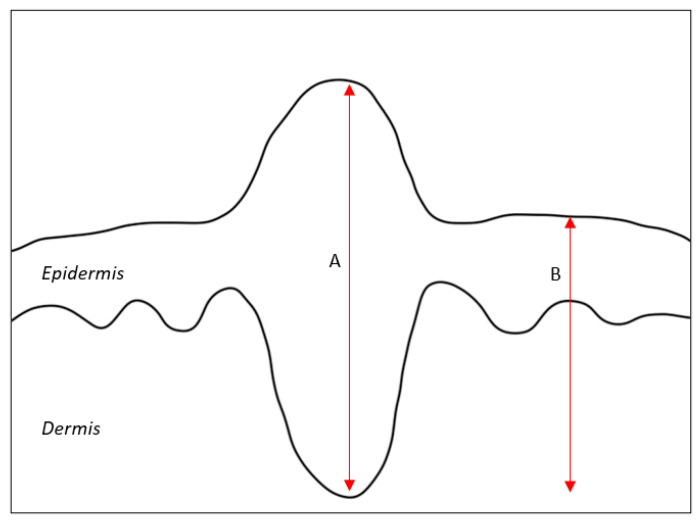
Measuring DOI with (A) Breslow depth or (B) measurement from top of adjacent, uninvolved granular layer as recommended by AJCC.

Depth of invasion (DOI) is widely recognized as a poor prognostic factor for cSCC of the head and neck. In a 2016 systematic review, DOI greater than 2 mm was associated with the highest relative risk of local recurrence and metastasis of head and neck cSCC when compared with other pathological features such as tumor diameter and differentiation, among others [4]. Further reviews have demonstrated that DOI is the most significant factor impacting prognosis for this cancer [2]. At that time, the 7th edition of the AJCC staging system defined DOI by either the Breslow depth or invasion beyond subcutaneous fat (anatomic depth) [5]. The authors found no difference in the relative risk of local recurrence and metastasis when comparing these two definitions of DOI. However, while the 8th edition of the AJCC staging system still includes anatomic depth as a way to measure DOI, the AJCC now also defines DOI as tumor invasion greater than 6 mm from the adjacent uninvolved granular layer to the tumor base, rather than as the Breslow depth (Figure 1) [6].

In a 2020 study, Yildiz et al. compared both Breslow depth and the new method of measuring DOI in the 8th edition of the AJCC staging system [7]. They found that Breslow depth measurement had a higher correlation with recurrence-free survival but that the AJCC recommendation was markedly correlated with recurrence-free survival when the cutoff depth was 8.7 mm rather than 6 mm. Measuring DOI by anatomic depth yielded results similar to those for Breslow depth in predicting recurrence-free survival. The authors suggested standardizing DOI measurements to exclusively use anatomic depth, noting that both the AJCC and Breslow measurement methods rely on a uniform thickness measurement even in tissues that vary widely in granular layer thickness. For example, a tumor with a DOI of 6 mm is considered high-risk whether it is found on the malar surface or upper eyelid, even though a 6 mm invasion into the eyelid represents a far greater disease burden than a 6 mm invasion into the malar surface [4].

Furthermore, authors reported tumor depth beyond the dermis as the most important risk factor for incomplete primary tumor excision. This results in an increased risk of recurrence, progression, and metastasis [8]. Ultimately, more research on clinical outcomes of cSCC based on the definition of DOI in both the 8th and the upcoming 9th editions of the AJCC staging system is warranted given its significant impact on recurrence and progression.

The role of DOI in determining the need for adjuvant therapy for cSCC remains ambiguous. In 2020, Ruiz et al. reported that for cSCCs with negative margins and no nodal metastasis, the effects of adjuvant radiotherapy (ART) with surgical excision on disease-specific survival, nodal or distant metastasis, and local recurrence (LR) were not markedly different from those of surgery alone [9]. This persisted even when the authors risk-stratified patient groups based on DOI beyond subcutaneous fat. In another study of patients with high-risk cSCC, defined by either the presence of desmoplasia (including PNI) or DOI greater than 6 mm, all patients underwent ART. The investigators observed no differences in LR rates for tumors with DOI less than 6 mm and those with DOI greater than 6 mm. However, radiation doses less than 60 Gy were closely associated with higher LR rates than were doses greater than 60 Gy [10].

The current NCCN guidelines are flexible regarding treatment of cSCC based on DOI. Of note, greater than 6 mm DOI or invasion beyond subcutaneous fat is considered “very-high-risk” disease, but guidelines still advise surgical resection if feasible. The NCCN further suggests multidisciplinary evaluation and consideration of adjuvant therapy [3]. Randomized comparative studies are needed to definitively determine the optimal treatment.

## 3. Surgical Margins

Incomplete resection of cSCC is associated with reduced recurrence-free and overall survival durations and an increased risk of metastasis and LR [11,12]. Furthermore, incompletely excised cSCCs may be more likely than completely excised cSCCs to progress histologically to a higher grade, as reported by Spyropoulou et al. [13]. The standard approach for a tumor with positive margins is re-excision when feasible, which improves prognosis. A systematic review of incompletely excised cSCCs demonstrated an LR rate of 5% when re-excision yielded negative margins, which was comparable with LR rates after initial excision with negative margins [14].

However, re-excision of cSCC may not be feasible, especially in the head and neck region, where the complex anatomy and subsequent morbidity may prevent further surgery. In such cases, ART is the treatment of choice [8,12]. The role of systemic therapy in these cases is reserved only as a supplement to ART, as chemotherapy alone is not indicated except for palliative purposes when surgery or ART are infeasible [15]. Advances in immunotherapy have yielded anti-PD-1 agents as efficacious treatment options for locally advanced cSCC [16,17]. However, their role in adjuvant treatment accompanying resection with positive margins has yet to be extensively studied.

Because complete surgical resection of cSCC is potentially curative while positive margins can necessitate further treatment, the utility of intraoperative frozen pathology must be carefully considered. A 2015 retrospective study of head and neck cSCCs demonstrated a discrepancy between frozen and permanent section margins in 20% of the cases studied [18]. Although the difference was not significant because of a small sample size (*n* = 41), this still represents a clinically relevant shortfall of relying on frozen sections for accurate margin assessment. A discrepancy between frozen and permanent margin assessment in that study was more likely to occur if the primary tumor had certain high-risk features, such as PNI or lymphovascular invasion (LVI). This points to the utility of a pre-resection biopsy to determine which high-risk features of cSCC are present before definitive resection and to the importance of careful surgical planning, particularly if the defect left from resection could be cosmetically unfavorable or require reconstruction.

The current NCCN guidelines do not have specific margin recommendations for high-risk SCCs, which includes all cSCCs of the head and neck. The NCCN emphasizes assessment of subclinical extension, lesion location, and presence of other high-risk features such as PNI in guiding margin assessment during resection. Consistent with the literature, the NCCN recommends re-excision or ART if resection yields positive margins. Systemic therapy is recommended only if ART is not feasible, with immunotherapy being the regimen of choice [3].

## 4. Positive Regional Node(s) and Extranodal Extension

Nodal metastasis of cSCCs of the head and neck is well known to portend poor prognosis, affecting overall survival, disease-specific survival, and time to recurrence [19,20,21]. Furthermore, tumor extension beyond the lymph node capsule (extranodal extension [ENE]) may also be a negative prognostic indicator for head and neck cSCC, as it is significantly associated with treatment resistance and tumor recurrence [22,23]. However, ENE alone may not independently result in unfavorable outcomes. The actual number of involved lymph nodes can determine the importance of ENE; survival outcomes are better when ENE is present with only one positive lymph node as opposed to ENE with multiple positive nodes, which worsens prognosis [24]. Also, the significance of ENE alone as a prognostic factor may be debatable from a tumor biology perspective; soft tissue metastasis overall may be a more accurate prognosticator, given its independent ability in predicting survival in comparison to ENE alone [25].

The consensus among reports in the literature is that nodal disease does not necessarily require ART, though it is reported to improve outcomes [19,20,21,26]. Therefore, it may be pertinent to consider the use of ART with nodal disease regardless of severity or feasibility of lymph node dissection; current NCCN guidelines allow for observation if nodal disease is limited to one node less than 3 cm without ENE. However, the literature consistently reports that the presence of ENE necessitates ART, which is also in line with the NCCN guidelines [12,15,16].

Furthermore, in some institutions outside the United States, ENE is an indication for systemic therapy. The authors of the cited study did not specify the drugs used; nevertheless, it is not common practice [27]. In the United States, systemic therapy for ENE of cSCC is generally reserved for cases in which ART is not feasible, with first-line treatment consisting of anti-PD-1 immunotherapy [15].

**Figure 2:**
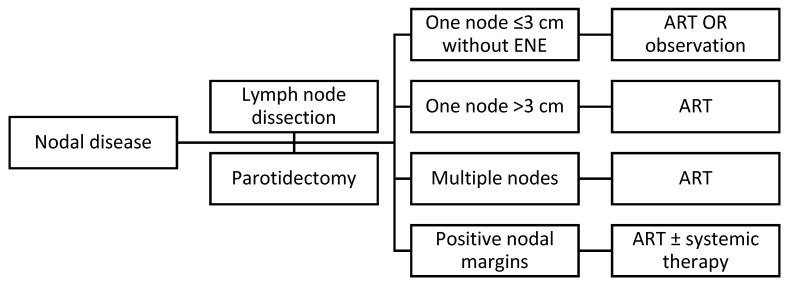
Treatment schematic for nodal disease in cSCC.

As described above, the NCCN guidelines recommend ART for tumors with ENE. Furthermore, the guidelines also suggest consideration of concurrent systemic therapy. Of note, this is specified for any node that is found to have ENE, whether unilateral or bilateral [3]. A treatment algorithm summarizing management for nodal disease can be found in Figure 2.

## 5. PNI

PNI is strongly associated with poor prognosis for head and neck cSCC [4,11,12,15]. However, several factors can further stratify the impact of PNI on prognosis. PNI with radiographic correlation of nerve involvement, cranial nerve symptoms, and PNI of named nerves portend worse outcomes than do PNI incidentally discovered in pathological examination and PNI of unnamed nerves [28].

**Figure 3:**
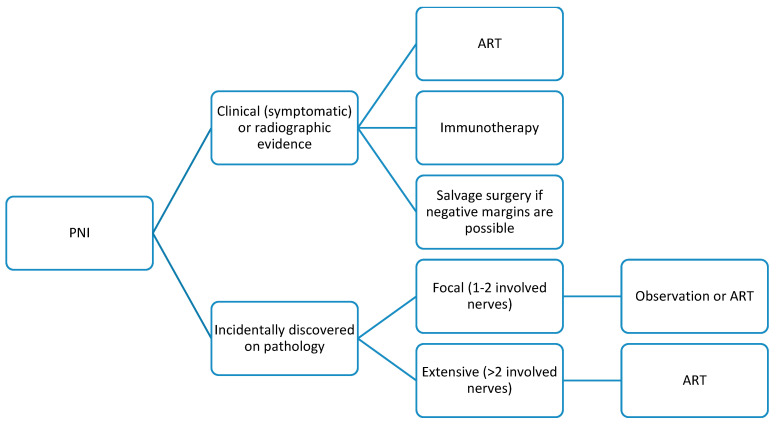
Possible treatment schematic for primary cSCC with PNI.

ART is generally favored as an adjuvant treatment of cSCC with PNI in the literature. However, some of the literature suggests that the extent of PNI may further clarify the need for ART. In 2016, Sapir et al. found that when stratifying microscopic PNI into categories deemed “focal” (1–2 involved nerves) and “extensive” (>2 involved nerves), ART did not have a greater effect on disease-free survival (DFS) when compared with observation alone in patients with focal PNI [29]. Furthermore, immunotherapy has been associated with improved DFS and radiographic disease control in patients with tumors with PNI. In a case series of 11 patients who underwent immunotherapy for cSCC with PNI, Wu et al. reported a sustained disease control rate of 82% [30]. They additionally found that neuropathic pain improved in patients receiving immunotherapy for clinical PNI. Although this was not a comparative study, the benefit of immunotherapy for cSCC with PNI should be considered by clinicians, especially for patients suffering from severe pain. Salvage surgery also can be considered for clinical PNI with skull base involvement if negative margins can be obtained. The impact of salvage surgery on DFS in cSCC with PNI varies based on the extent of involvement, particularly if cranial nerve invasion complicates salvage surgery. However, when feasible, salvage surgery combined with irradiation can improve DFS, though again, comparative studies are lacking [31]. A potential treatment algorithm summarizing these findings can be found in Figure 3.

The NCCN guidelines recommend that clinicians consider ART for cSCC with PNI and also state that if cranial nerve symptoms or PNI of named nerves is present, the courses of the involved nerves should be incorporated in planning ART [3]. Because PNI automatically classifies any cSCC as T3 disease, the NCCN does not provide treatment recommendations based on both PNI and stage, as PNI is considered staging information in itself.

## 6. LVI

Defined as tumor cell presence in the endothelium of a lymphatic or vascular lumen, LVI of cSCC is an indicator of aggressive disease. In a 2022 retrospective study, Farah et al. reported that LVI was highly associated with poor overall survival and disease-specific survival [32]. Also, in an analysis of 4252 cSCCs of the head and neck, Kus et al. reported a negative effect of LVI on distant metastasis and disease-specific survival in patients with tumors of all stages [33].

LVI in primary cSCC specimens was reported to be a significant indicator of nodal disease, suggesting the importance of sentinel lymph node biopsy analysis of these tumors [19,34]. If the presence of LVI indicates nodal disease, treating cSCCs with LVI as such—namely, with ART—would be reasonable [26]. However, nodal disease should first be confirmed with sentinel lymph node biopsy or neck dissection; therefore, LVI in a tumor specimen should prompt further nodal involvement workup. The location of the primary tumor must also be considered. Kadakia et al. reported that for cSCC in the temporal region, tumors greater than 2 cm in diameter with parotid disease were frequently associated with LVI [35]. The authors thereby highlighted the role of superficial parotidectomy when LVI of temporal region tumors is found.

Similar to those regarding PNI, the NCCN guidelines do not definitively require ART for cSCC with LVI, only suggesting careful consideration by clinicians. However, it does suggest the use of immunotherapy for LVI in “very high-risk” disease, such as desmoplastic or poorly differentiated tumors. Notably, LVI is not included as a pathological trait of cSCC in the 8th edition of the AJCC staging system, despite being a recommended part of diagnostic pathology reports by the NCCN and a consistently documented indicator of poor prognosis for cSCC [3].

## 7. Tumor Grade

Histologically, cSCC can be categorized as well, moderately, or poorly differentiated. The degree of differentiation has been independently associated with several unfavorable clinical outcomes, including metastasis, poor overall survival, LR, and poor disease-specific survival [4,36].

Although the role of adjuvant treatment of cSCC based specifically on tumor grade has not been studied, poor differentiation of cSCC may complicate margin assessment during surgical excision, warranting further treatment [37]. Indeed, in 2020, Kiely et al. reported an increased rate of incomplete excision of poorly differentiated cSCCs despite consistently clearing the recommended peripheral margins of 6 mm [38]. Therefore, ART may be reasonable in select cases if the primary tumors are poorly differentiated; as described previously, positive margins can be indications for postoperative ART [8,12].

The NCCN guidelines classify poor differentiation as a marker of high-risk cSCC. However, the NCCN does not recommend a specific adjuvant treatment of poorly differentiated cSCC, only suggesting consideration of ART, particularly if other poor prognostic factors as defined by the 8th edition of the AJCC staging system are present. Yet, tumor grade was removed from the AJCC system due to inconsistent definitions of differentiation [3,39]. In 2018, Karia et al. conducted a review of 459 patients to compare the 7th and 8th editions of the AJCC staging system and examine any differences in clinical outcomes [40]. They found that for the tumors upgraded to stage T3 when switching from the 7th to the 8th AJCC edition system, unfavorable outcomes were largely associated only with poorly differentiated T3 tumors, not the well or moderately differentiated T3 tumors. Therefore, some well-to-moderately differentiated tumors may be inappropriately upgraded to stage T3, despite having better clinical outcomes than poorly differentiated T3 tumors.

## 8. Histologic Subtype

Distinct subtypes of cSCC, beyond the classic progression of premalignant lesions, exist and can influence disease prognosis. Clear cell carcinoma is an extremely uncommon variant of cSCC first described in 1980 by Kuo et al. [41]. Due to its rarity, its impact on prognosis is not well understood, with a variety of reported outcomes. The original 1980 study suggests a favorable disease course with aggressive surgical resection, even if the primary tumor was bulky or invasive. Further case reports document patients who were lost to follow-up but describe rapidly growing, locally destructive tumors [42,43]. Histopathological analysis in Kuo’s case series revealed extensive perineural and vascular invasion along with cervical nodal metastasis [41]. Therefore, owing to the paucity of literature, it may be beneficial to treat clear cell cSCCs as tumors with perineural and vascular invasion, the prognostic impact of which has been described earlier in this review.

Spindle cell carcinoma is another rare subtype of cSCC, first described in 1935. Similar to the clear cell variant, its rarity renders prognostic estimates difficult to make. Notably, its clinical course may be influenced by the underlying etiology—de novo spindle cell cSCC has a more indolent disease course than radiation-induced spindle cell cSCC [44]. Radiation-induced disease can be aggressive, with a higher likelihood of metastasis and death. However, depth of invasion is likely a more important prognostic factor in radiation-induced spindle cell cutaneous carcinoma, given the lack of robust literature describing its prognostic indicators [45]. The aggressive disease course may be related to its poor differentiation on histology. In any case, surgical excision is the recommended treatment [46]. NCCN characterizes it as a high-risk pathology; however, it may be reasonable to first determine the lesion’s DOI and size, the prognostic implications of which have been discussed in Section 2 of this review.

Verrucous carcinoma (VC) of the skin is another rare subtype of cSCC. Its association with human papilloma virus types 6 and 11 is not well understood and is described inconsistently in the literature [47]. Verrucous cancer itself can be divided into subtypes based on anatomic origin, including the oral mucosa, anogenital region, and more rarely, primary cutaneous lesions [48]. Head and neck tumors are relatively uncommon areas for VCs. These tumors are slow-growing and very rarely metastasize, though they can be locally destructive; the treatment of choice is surgical excision [49]. NCCN characterizes VC as a low-risk histologic subtype of cSCC, affirming surgical excision as the definitive treatment [3].

Lymphoepithelioma-like carcinoma of the skin (LELCS) is a subtype of cSCC that is histologically identical to nasopharyngeal lymphoepithelioma, though LELCS is not associated with Epstein–Barr virus [50]. LELCS is considered a low-risk cSCC variant, with only two reported deaths in the literature [51]. As such, treatment is straightforward, with surgical excision as the mainstay; ART may be employed for inoperable or recurrent tumors [52]. One case report of LELCS documents the use of ART due to observed PNI on histopathology, the prognostic and treatment implications of which have been discussed earlier in this review [51]. LELCS is not mentioned in NCCN guidelines and therefore does not have definitive treatment recommendations.

Desmoplastic cSCC is considered a high-risk histologic subtype. First described in 1989 and fully characterized in 1997, desmoplastic cSCC has been associated with poorer tumor differentiation, local recurrence, metastasis, and worse overall survival [53]. In fact, desmoplastic cSCC has been found to be an independent predictor of local recurrence despite adequate resection with negative margins [54]. Therefore, patients with desmoplastic cSCC should be closely monitored even after successful resection due to their elevated risk of disease-related death. Ultrasound of regional lymph nodes and sentinel lymph node biopsy have both been suggested as possible surveillance tools in this population [54,55]. The NCCN guidelines categorize desmoplastic cSCC as “very high risk” and recommend the consideration of ART or neoadjuvant cemiplimab for such cases.

## 9. Premalignant Lesions

The vast majority of cSCCs represent histologic transformation of premalignant lesions such as actinic keratoses (AKs). AKs are the most common premalignant skin lesions, with over 40 million diagnoses in America alone per year [56]. While the overall risk of transformation of individual AKs to invasive cSCC is low, certain clinical and pathologic variables carry worse prognosis. They typically present as scaly, erythematous patches or papules with ill-defined margins. High-risk clinical features include size >1 cm, bleeding, ulceration, induration, or rapid growth of a lesion [57,58]. The presence of any of these features on exam should prompt biopsy to exclude invasive cSCC. However, low-risk clinical lesions can still have high-risk histology; specifically, the proliferative and hypertrophic AK subtypes are more aggressive and more likely to progress to cSCC [59]. Therefore, all AKs should be treated when diagnosed, even if lesions appear clinically benign.

AKs are treated topically, with several well-established options. The approach to treatment can either be lesion-based or field-based, depending on lesion or patient characteristics. Lesion-based treatments include scalpel excision, cryotherapy, and laser. Field-based treatments are useful in patients with multiple contiguous lesions or widespread ultraviolet damage that could conceal subclinical lesions. Many topical agents can be used, including, but not limited to, topical 5-fluorouracil, diclofenac, or imiquimod. Photodynamic therapy combined with other topical agents may also be used as a field-based AK treatment [60,61].

Bowen’s disease, considered cSCC in situ, is another common precursor lesion to invasive cSCC. Similar to AK, the risk of malignant transformation is low, estimated at between 3 and 5% [62]. However, the potential for metastasis upon transformation to invasive disease can be as high as 20%, making prompt diagnosis and treatment critical [59]. These lesions typically present as well-circumscribed, erythematous, hyperkeratotic plaques with scaling. Features that are associated with invasive disease and thus should prompt biopsy include size > 10 mm, ulceration, bleeding, or nodule formation [63,64].

Treatment options for cSCC in situ are similar to that of AK, ranging from topical therapy to curettage or excision. Nonsurgical therapy may be the superior modality, and photodynamic therapy specifically is cosmetically favorable to topical agents such as 5-fluorouracil with no significant difference in response to treatment [65]. Furthermore, unlike AK, radiotherapy may also be utilized as a treatment option, particularly for unresectable lesions or those that do not respond to topical agents [66].

Current NCCN guidelines recommend treatment for both AKs and cSCC in situ due to their risks of malignant transformation. For AKs, NCCN recommends topical 5-fluorouracil as a first-line treatment, citing more favorable progression-free survival compared with imiquimod or photodynamic therapy [3,67]. Consistent with the literature, NCCN recommends nonsurgical management of cSCC in situ, with the main options being 5-fluorouracil, imiquimod, and photodynamic therapy. However, the guidelines caution that the efficacy of photodynamic therapy may be widely impacted by technique [3].

## 10. Molecular Markers

Various molecular markers found in primary cSCCs, elucidated through immunohistochemistry, can reflect underlying tumor biology. These markers can predict tumor behavior, prognosis, and provide specific targets for treatment. One proposed mechanism of metastasis in cSCC, known as epithelial–mesenchymal transition (EMT), involves de-differentiation of single tumor cells upon detaching from the primary lesion. Proteins involved in cellular adhesion, namely E-cadherin, must be downregulated to allow EMT. Indeed, downregulation of E-cadherin has been observed in primary cSCCs, particularly in lymph node metastases and poorly differentiated tumors [68]. However, E-cadherin under-expression does not seem to be a predictor of distant metastasis, suggesting EMT as only a partial explanation for cSCC metastasis [68]. Therefore, the under-expression of E-cadherin may be particularly useful in predicting degree of differentiation, which in itself can portend a higher likelihood of metastasis, LR, or disease-specific survival, as previously discussed [68].

Another hypothesis for cSCC metastasis posits collective cancer invasion, which relies on groups of adherent cells that can separate from the primary tumor, as opposed to the singular cells proposed by the EMT theory. Collective cancer invasion has been implicated in the tumorigenesis of other cancers, such as prostate and pancreatic cancers [68]. This mechanism requires a number of cells in the group of adherent cells to guide the rest, necessitating the use of cytoskeletal proteins such as podoplanin. Its role in predicting metastatic disease has been established in other squamous cell carcinomas, including those of the esophagus and lung [69]. Several studies have demonstrated that increased podoplanin expression in primary cSCCs is associated with nodal metastasis and worse overall survival compared to tumors with low podoplanin expression [68,70]. Its use in predicting distant metastasis, however, has not been consistent in the existing literature for cSCC [68,69].

A key factor in cSCC tumorigenesis is immune downregulation; upregulation of programmed death ligand-1 (PD-L1) by tumor cells is a well-established mechanism of evading the host immune response in many cancers, cSCC included. High expression and staining of PD-L1 in primary cSCC tissue is associated with higher risk of both lymph node and distant metastasis [69,71,72]. However, high expression of PD-1 in itself does not determine treatment modality; rather, it should prompt careful evaluation for nodal and distant metastasis and be treated according to the AJCC stage. The use of immune checkpoint inhibitor and anti-PD-L1 therapy is reserved for locally advanced, recurrent, or metastatic disease, not necessarily for tumors with high PD-L1 burden.

Similarly, epidermal growth factor receptor (EGFR) is also a well-known marker of cellular proliferation, and its overexpression has been implicated in cSCC pathogenesis. While some of the literature indicates no association with aggressive disease or survival in cSCC, recent studies demonstrate that EGFR overexpression carries a higher likelihood of nodal metastasis, distant metastasis, and TNM progression [73,74,75]. Its importance lies in the availability of targeted therapy via cetuximab, though like anti-PD-L1 therapy, its use is for metastatic or inoperable disease.

Neither the current literature nor NCCN guidelines recommend any specific treatment based on molecular markers or immunohistochemical staining. However, the presence of such markers may be predictive of nodal or distant metastasis, and patients should undergo careful evaluation and treatment based on AJCC stage. A summary of recommendations and treatment modalities can be found in Table 1 and Table 2.

## 11. Conclusions and Future Directions

Many pathological factors impact prognosis for cSCC in the head and neck. While current treatment guidelines by the NCCN are undoubtedly informative and highly evidence-based, particularly for factors with robust supporting literature, other pathologic factors provide crucial disease information. Given the disease’s prevalence and potential for aggressive disease in the presence of such factors, further work regarding prognosis is needed in several areas.

First, the AJCC staging system or NCCN guidelines should provide a standardized definition and conceptualization of features such as DOI and PNI, namely because slight definitional modifications of DOI and PNI can alter risk stratification and therefore treatment regimens in these patients. This is particularly important when considering DOI because the skin granular layer may differ in thickness based on location on the head and neck, making depth by millimeter a potentially unreliable metric. Furthermore, researchers ought to continue to investigate the role of adjuvant therapy for these tumors given the presence of pathologic prognostic features as described in this review, including irradiation, immunotherapy, and systemic therapy. Finally, more investigation of pathological variants of cSCC, such as desmoplastic, adenosquamous, and basosquamous carcinomas, is needed.

## Figures and Tables

**Table 1:** Summary of pathologic features and associated treatment recommendations.

Feature	Threshold	Recommendations
DOI	6 mm from adjacent uninvolved granular layer	No specific therapy recommendations
	Invasion beyond subcutaneous fat	
Margins	Positive	Re-excision if feasible, ART ± systemic therapy if not
LVI	Present	Evaluate for nodal disease and treat appropriately
ENE	Present	ART ± systemic therapy
PNI	Clinical or radiographic	ART
	Microscopic	ART vs. observation
Grade	Poor differentiation	Evaluate margins and treat appropriately
Subtype	Desmoplastic	ART vs. neoadjuvant cemiplimab
Molecular markers	PD-L1, Podoplanin, EGFR	Evaluate for nodal/distant metastasis and treat appropriately

**Table 2:** Summary of pathologic indications for treatment modalities.

Treatment Options	Indicated Feature(s)
ART	PNI
	Positive margins with unfavorable resection
	Multiple lymph nodes or one node >3 cm
	ENE
	LVI
	Poorly differentiated primary tumor
	Desmoplastic subtype
Re-operation	Positive margins
	PNI if adequate margins are feasible
Immunotherapy	PNI
	Desmoplastic subtype
Other systemic therapy	Only as ART adjunct or if ART is infeasible
